# Assessing preventive health behaviors from COVID-19: a cross sectional study with health belief model in Golestan Province, Northern of Iran

**DOI:** 10.1186/s40249-020-00776-2

**Published:** 2020-11-17

**Authors:** Hossein Shahnazi, Maryam Ahmadi-Livani, Bagher Pahlavanzadeh, Abdolhalim Rajabi, Mohammad Shoaib Hamrah, Abdurrahman Charkazi

**Affiliations:** 1 Department of Health Education and Promotion, School of Health, Isfahan University of Medical Sciences, Isfahan, Iran; 2 Faculty of Health, Golestan University of Medical Sciences, Gorgan, Iran; 3 Department of Biostatistics, School of Allied Medical Sciences, Shahid Beheshti University of Medical Sciences, Tehran, Iran; 4 Faculty of Health, Environmental Health Research Center, Golestan University of Medical Sciences, Gorgan, Iran; 5 Center for Rural Health, School of Health Sciences, University of Tasmania, Hobart, Australia; 6 Faculty of Health, Environmental Health Research Center, Late Falsefi University Complex, KM 5of Gorgan-Sari Road, Golestan, Iran

**Keywords:** COVID-19, Health belief model, Fatalism, Preventive behavior, Iran

## Abstract

**Background:**

Coronavirus disease 2019 (COVID-19) is a new viral disease that has caused a pandemic in the world. Due to the lack of vaccines and definitive treatment, preventive behaviors are the only way to overcome the disease. Therefore, the present study aimed to determine the preventive behaviors from the disease based on constructs of the health belief model.

**Methods:**

In the present cross-sectional study during March 11–16, 2020, 750 individuals in Golestan Province of Iran were included in the study using the convenience sampling and they completed the questionnaires through cyberspace. Factor scores were calculated using the confirmatory factor analysis. The effects of different factors were separately investigated using the univariate analyses, including students sample *t*-test, ANOVA, and simple linear regression. Finally, the effective factors were examined by the multiple regression analysis at a significant level of 0.05 and through Mplus 7 and SPSS 16.

**Results:**

The participants’ mean age was 33.9 ± 9.45 years; and 57.1% of them had associate and bachelor's degrees. Multiple regression indicated that the mean score of preventive behavior from COVID-19 was higher in females than males, and greater in urban dwellers than rural dwellers. Furthermore, one unit increase in the standard deviation of factor scores of self-efficacy and perceived benefits increased the scores of preventive behavior from COVID-19 by 0.22 and 0.17 units respectively. On the contrary, one unit increase in the standard deviation of factor score of perceived barriers and fatalistic beliefs decreased the scores of the preventive behavior from COVID-19 by 0.36 and 0.19 units respectively.

**Conclusions:**

Results of the present study indicated that female gender, perceived barriers, perceived self-efficacy, fatalistic beliefs, perceived interests, and living in city had the greatest preventive behaviors from COVID-19 respectively. Preventive interventions were necessary among males and villagers.

## Background

On January 30, 2020, The World Health Organization's Emergency Committee considered it as a global health emergency due to its significant growth, and declared it to be a pandemic in March 2020 [1].

SARS-CoV-2 has a genetic similarity of 96% to corona virus originated from bats [2]. Early symptoms of SARS-CoV-2 are related to coronavirus disease 2019 (COVID-19) that occurs with pneumonia symptoms. Recent reports indicate gastrointestinal symptoms and asymptomatic infections, especially in children [3]. Its incubation period is with mean of 5 days and median of 3 days and a range of 0–24 days [2, 4]. Clinical manifestations of the disease usually occur within less than a week. The symptoms include fever, cough, nasal inflammation, fatigue, and other signs of upper respiratory tract infection [4].

A feature of the SARS-CoV-2 is its high virulence. Results of the recent study on 425 patients indicated that the number of patients doubled per week in the current pandemic; and each patient infected 2.2 individuals on average [5]. Analysis of recent results from the early stages of the outbreak also indicated that the rate ranged from 2.2 to 3.58 individuals [6].

In Iran, the first case was reported in Qom on February 19, 2020, and then it spread to other regions of Iran. Until April 10, 2020, 66 220 individuals had the disease and 4110 died in Iran [7]. The disease has been reported in 197 countries so far; and 1 521 252 cases of coronavirus were reported worldwide until April 10, 2020 according to Johns Hopkins University. 92 798 of the patients died from the disease. Iran ranks sixth after China, Italy, the United States, Spain and Germany [8].

No vaccine or definitive treatment has been found for the disease so far, and the treatments are symptomatic and supportive. Washing hands regularly with soap and water, covering mouth and nose when coughing and sneezing, and not touching the nose, mouth and eyes, wearing face masks, social distancing and good ventilation are the only ways to prevent the spread of COVID-19 [9]. Each person is the most important factor in promoting health; and the right or wrong behaviors are influenced by the individuals' beliefs, values, tendencies, and habits [5]. Sociologists, psychologists, and anthropologists have proposed a range of different theories and models to explain the factors influencing the health behavior, one of which is the health belief model (HBM). This model is introduced by Rosenstock et al. and is a general conceptual framework and theoretical guideline for health behaviors in the public health research, and it consists of constructs, namely the perceived susceptibility, perceived severity, perceived benefits, perceived barriers, cues to action, and preventive health behaviors [10, 11]. The general acceptance and popularity of the health belief model is due to its high predictive power [11]. The model is designed to explain the reasons why people do not participate in the prevention program and is based on the hypothesis that the individuals' preventive behavior is affected by their beliefs in being at risk (perceived susceptibility), the seriousness of risk (perceived severity), existence of a way to reduce the incidence or severity of disease (perceived benefits), and higher costs versus the benefits of action (perceived barriers), and thus they participate in screening and prevention activities based on the evaluation of these factors [12, 13]. During the outbreak of COVID-19 pandemic, large epidemiological study found that high frequency of wearing masks regardless of the presence or absence of symptoms was significantly associated with lower anxiety and depression levels [14]. Better hygiene practices and avoidance of sharing utensils during meals were significantly associated with lower psychological impact, depression, anxiety and stress at outbreak and 4 weeks after the outbreak [15].

Given the pandemic and spread of SARS-CoV-2, the observance of preventive health standards and behaviors in society is essential to better control the disease. Therefore, the present study was conducted to determine the preventive health behaviors from COVID-19 based on the health belief model among people in Golestan Province in March 2019.

## Methods

### Procedure

The present cross-sectional study was conducted in a population of over 18 years of age in Golestan Province in northern Iran from March 11 to 16, 2019. The participants were selected using the convenience sampling and they completed the electronic questionnaire. Generalities of the research were approved in Research Council of Golestan University of Medical Sciences and the National Ethics Committee in Biomedical Research with a code of IR.GOUMS.REC.1398.384. The questionnaire was forwarded via the virtual networks in Telegram and WhatsApp groups and channels, and the individuals were asked to optionally complete it and forward it to their friends and acquaintances. The approximate time to complete the questionnaire was about ten minutes, and it first started with an explanation of the research objectives.

### Measures

The research tool included a questionnaire consisting of five sections, including demographic questions, health belief model construct questions, fatalism questions, clinical symptom recognition questions, and questions on the preventive behaviors from COVID-19.

1. Demographic questions: The section provided questions about age, gender, education, place of residence (city or village) and city of residence.
2. Questions about health belief model constructs, including six sections (questions about perceived susceptibility (three questions), perceived severity (three questions), perceived benefits (three questions), perceived barriers (eight questions), sense of self-efficacy (one question), and cues to action (two questions).
3. The fatalism section included two questions. All construct questions of the health belief model and fatalism were on a 5-point Likert scale (from strongly agree to strongly disagree), and their scores ranged from 1 to 5.
4. Questions about recognizing the clinical symptoms of disease (seven questions) that were answered by yes, no and I don't know. The correct answer was scored 1, the wrong answer and I don't know were scored 0.
5. There were eight questions about the preventive behaviors from COVID-19. Answering the questions was on a 5-point Likert scale from Always to Never; and scoring was from 1 to 5.

The opinions of 8 health education and promotion specialists were used to determine the content validity; and the necessary changes and corrections were applied in the text of the questionnaire based on their opinions. Besides, the confirmatory factor analysis indicated that the measures have in acceptable ranges (Table 1).

**Table 1:** Confirmatory factor analysis values regarding to study measures

RMSEA (95% confidence interval)	CFI	TLI	SRMR	
0.056 (0.046–0.066)	0.916	0.893	0.06	Susceptibility
0.047 (0.037–0.058)	0.925	0.905	0.04	Severity
0.055 (0.049–0.081)	0.914	0.901	0.04	Barriers
0.048 (0.036–0.06)	0.94	0.921	0.039	Benefits
0.043 (0.031–0.055)	0.948	0.931	0.036	Fatalism
0.053 (0.041–0.066)	0.929	0.906	0.042	Self-efficacy
0.049 (0.037–0.061)	0.933	0.911	0.039	Cues to action
0.05 (0.41–0.077)	0.908	0.905	0.051	Clinical symptom

*RMSEA* root mean square error of approximation, *CFI* comparative fit index, *TLI* Tucker Lewis index, *SRMR* standardized root mean square residual

### Data analysis

First, the data were included in Mplus Version 7.2. (Los Angeles, CA: Muthén & Muthén). Thereafter, the confirmatory factor analysis was used to investigate the relationship between each variable of the health belief model and fatalistic behaviors with individual behaviors about the prevention of COVID-19 for each variable, and then the desired structural models about relationships of each variable with the performance were inserted in the software. In each of these factor analyses, the factor score was calculated and stored for each variable. In the analyses, the goodness of fit indices, including the Root Mean Square Error of Approximation (RMSEA), Comparative fit index (CFI), Tucker Lewis index (TLI), and Standardized Root Mean Square Residual (SRMR) were used to judge the suitability of model. The Mann–Whitney test and Spearman's correlation analysis were relatively used to investigate the association between gender, residence place, and age with preventive behaviors from COVID-19. The factor scores in the simple linear regression analysis were used to investigate the effect of each construct on the performance of COVID-19 prevention behavior, and finally, effects of all constructs were examined simultaneously on COVID-19 preventive behavior using the multiple linear regression. The analyses were performed at a significance level of 0.05 using the SPSS for Windows, Version 16.0. Chicago, SPSS Inc.).

## Results

### General findings

Participants (*n* = 750) were in the age range of 15–77 with an average age of 33.9 ± 9.45 years. 394 individuals (52.5%) were male, 74.9% lived in cities, and 57.1% had associate and bachelor's degrees. The mean scores of preventive behaviors from COVID-19 indicated significant differences with gender and residence place (Table 2).

**Table 2:** Frequency distribution of demographic variables of the participants

Variable	Group	Number (%)
Gender	Male	394 (52.5)
	Female	356 (47.5)
Residence place	Rural	188 (25.1)
	Urban	562 (74.9)
Education	Under high school diploma	74 (9.9)
	High school diploma	109 (14.5)
	Associate and bachelor's degree	428 (57.1)
	Master and higher	139 (18.5)

The research results indicated that most participants (96.8%) did not go to crowded places due to the prevention of the disease. 54% believed that people follow hygienic standards such as using masks, and hand washing to prevent the disease, while 25.2% believed that people never observe hygiene standards.

The results indicated that most respondents had relatively high perceived susceptibility, perceived severity, perceived benefits, and perceived self-efficacy, but lower perceived barriers and fatalistic beliefs (Table 3).

**Table 3:** Frequency distribution of answers to questions based on the fatalistic beliefs and health belief model constructs

Variable	Strongly agree Percent, No.	Partially agree Percent, No.	No idea Percent, No.	Partially disagree Percent, No.	Strongly disagree Percent, No.
Perceived susceptibility					
1. I consider myself to be at risk of coronavirus	40.7, 305	29.6, 222	9.7, 73	10.1, 76	9.9, 74
2. I am more likely to get the disease	24.8, 186	31.2, 234	15.5, 116	17.6, 132	10.9, 82
3. I don't care about this disease and do my daily activities like before	4.1, 31	6.1, 51	2.9, 22	16.7, 125	69.5, 521
Perceived severity					
1. This disease has a high mortality rate	33.9, 254	32.4, 243	8.9, 67	17.9, 134	6.9, 52
2. This disease is not very dangerous	3.5, 26	19.1, 143	4.9, 37	25.9, 194	46.7, 350
3. The transmission power of this disease is high	93.7, 7.1	4.9, 37	0.9, 7	0.3, 2	3, 0.4
Perceived barriers					
1. It is difficult to follow the instructions to prevent this disease	13.9, 104	31.7, 238	2.8, 21	23.1, 173	28.5, 214
2. I don't have the patience to follow preventative instructions	1.1, 8	8.5, 64	4.1, 31	22.4, 168	63.9, 479
3. It is difficult to wash hands regularly with soap and water	6, 45	16.9, 127	3.3, 25	20.7, 155	53.1, 398
4. The mask is scarce in the market, and thus I do not wear a mask	22.8, 171	23.1, 173	14.5, 109	17.7, 133	21.9, 164
5. Disinfectant gels and solutions are scarce and expensive in the market	59.3, 445	22.1, 166	9.6, 72	4.5, 34	4.4, 33
6. Alcohol pads are scarce in the market	54.4, 408	22.8, 171	15.5, 116	4.1, 31	3.2, 24
7. It is difficult not to touch hands, mouth, nose and eyes	20.1, 151	37.6, 282	4.2, 32	18.7, 140	19.3, 145
8. Staying at home to prevent the disease is difficult	22.7, 170	31.9, 239	4.7, 35	15.5, 116	25.3, 190
Perceived self-efficacy					
I have ability to follow every preventive instructions against the disease	43.1, 323	40.5, 304	5.5, 41	7.9, 59	3.1, 23
Perceived benefits					
1. This disease can be easily prevented by washing hands regularly with soap and water	45.1, 338	41.9, 314	4.7, 35	6.9, 52	1.5, 11
2. This disease can be easily prevented by personal protective equipment such as masks and disposable gloves	36.4, 273	48.7, 365	5.5, 41	7.3, 55	2.1, 16
Fatalistic beliefs					
1. Having this disease is bad luck and the prevention has no effect	1.6, 12	4.4, 33	5.3, 40	16.5, 124	72.1, 541
2. Catching or not catching the disease is out of my control	6.5, 49	15.2, 114	11.2, 84	32.7, 245	34.4, 258
Cues to action					
TV and radio information about the disease has been helpful	27.2, 204	28, 210	12.9, 97	11.5, 86	20.4, 153

The vast majority of samples were aware of three main symptoms of COVID-19, including fever, dry cough, and shortness of breath (Table 4).

**Table 4:** Frequency distribution of answers to questions of clinical symptoms of COVID-19

Questions	Yes No., Percent	No No., Percent	I don't know No., Percent
1. Is headache a main symptom of this disease?	273, 36.4	303, 4.4	174, 23.2
2. Is runny nose a main symptom of this disease?	211, 28.1	414, 55.2	125, 16.7
3. Is fever a main symptom of this disease?	701, 93.5	23, 3.1	26, 3.5
4. Is dry cough a main symptom of this disease?	717, 95.6	13, 1.7	20, 2.7
5. Is shortness of breath a main symptom of this disease?	731, 95.5	6, 0.8	13, 1.7
6. Are body and muscle pain the main symptoms of this disease?	451, 60.1	161, 21.5	138, 18.4
7. Are digestive problems (diarrhea and nausea) the main symptoms of this disease?	292, 38.9	277, 36.9	181, 24.1

### Preventive behaviors

Regarding preventive behaviors from COVID-19, 82% of participants "always" observed "no handshake and kissing", 73.7% observed "hand washing when entering the house", 64.3% observed "no need to leave the house", and 61.2% observed "the use of tissue paper or bending elbows when coughing and sneezing". 45.7% of samples always observed "washing hands with soap and water" and 39.7% always observed "a distance of one meter". The lowest levels of compliance were related to "touching face by hands" and "non-use of mobile phones outside the house", which were always observed by 33.5% and 22.8% of participants respectively (Table 5).

**Table 5:** Frequency distribution of conditions for observing preventive behaviors from COVID-19

Variable	Always No., Percent	Often No., Percent	Sometimes No., Percent	Rarely No., Percent	Never No., Percent
1. I place a tissue paper or bending elbow in front of my mouth and nose when coughing or sneezing	459, 61.2	245, 32.7	32, 4.3	11, 1.5	3, 0.4
2. I keep a distance of at least one meter from others	298, 39.7	352, 46.9	70, 9.3	25, 3.3	5, 0.7
3. I don't shake hands with others and don't kiss them	615, 82	102, 13.6	12, 1.6	4, 0.5	17, 2.3
4. I don't leave the house unless absolutely necessary	482, 64.3	190, 25.3	40, 5.3	23, 3.1	15, 2
5. I wash my hands regularly with soap and water for at least 20 s every hour	343, 45.7	270, 36	94, 12.5	31, 4.1	12, 1.6
6. I do not touch my eyes, nose and mouth by hands	251, 33.5	381, 50.8	78, 10.4	29, 3.9	11, 1.5
7. I do not take my cell phone out of my pocket	171, 22.8	264, 35.2	165, 22	110, 14.7	40, 5.3
8. I wash my hands with soap and water without touching anything after entering home	553, 73.7	165, 22	24, 3.2	5, 0.7	3, 0.4

### HBM constructs

The mean scores (standard deviation) for constructs of the health belief model and fatalistic beliefs presented in Table 6. The measured mean was obtained from dividing the mean score by number of questions in order to make the mean importance of each dimension comparable in participants. As presented, the "fatalistic beliefs" had the highest mean (4.13), followed by "perceived susceptibility" (3.03), "perceived barriers" (2.96), and "cues to action" (2.74). The lowest mean belonged to "perceived benefits" (1.83) and "preventive behaviors" (1.68) (Table 6).

**Table 6:** Frequency distribution of mean, standard deviation and standardized mean of fatalistic beliefs and health belief model constructs for preventive behaviors from COVID-19 in the participants

	Number of questions	Range of scores for questions	Mean	Sd.	Standardized mean	Minimum	Maximum
Perceived susceptibility	3	1–5	9.18	2.57	3.03	3	15
Perceived severity	3	1–5	7.34	1.41	2.41	3	15
Perceived barriers	8	1–5	23.7	6.11	2.96	8	40
Perceived benefits	2	1–5	3.68	1.62	1.83	2	10
Fatalistic beliefs	2	1–5	8.23	1.82	4.13	2	10
Perceived self-efficacy	1	1–5	1.87	1.03	1.87	1	5
Cues to action	2	1–5	5.48	2.05	2.74	2	9
Recognition of clinical symptoms	7	0–1	4.4	1.49	0.62	0	7
Preventive behaviors	8	1–5	13.51	4.17	1.68	8	34

The univariate analysis indicated that the self-efficacy, barriers, benefits, fatalism, cues to action, gender, and place of residence had significant effects on preventive behaviors from COVID-19. Multiple regression also indicated that self-efficacy, barriers, fatalism, gender, and place of residence were associated with preventive behaviors from COVID-19, and only the "cues to action" variable lost its significance. In this regard, the self-efficacy and perceived benefits had positive relationships; in other words, the mean score of performance increased with their increase, but the perceived barriers and fatalistic beliefs had opposite relationships and decreased the mean score of performance. Furthermore, the mean score of preventive behaviors against COVID-19 was higher in women than men and also higher in urban residents than villagers. As shown in the "standardized estimation" column of the table for comparing the effects of variables on performance, the greatest impact belonged to gender. The "perceived barriers" variable had a greater effect on preventive behaviors from COVID-19 than fatalism; and the individual's self-efficacy had a greater effect on the preventive behaviors from COVID-19 than the perceived benefits (Table 7).

**Table 7:** Effects of constructs of the health belief model, fatalistic beliefs, and demographic variables on preventive behaviors from COVID-19

	Univariate analysis				Multivariate analysis			
	Estimation	Confidence interval	Standardized estimation	*P*-value	Estimation	Confidence interval	Standardized estimation	*P*-value
Perceived self-efficacy	0.12	0.1 to 0.14	0.37	< 0.001	0.005	0.03 to 0.06	0.22	< 0.001
Perceived susceptibility	− 0.016	− 0.03 to 0.001	− 0.06	0.067	− 0.006	− 0.02 to 0.01	− 0.02	0.45
Perceived severity	− 0.02	− 0.02 to 0.06	0.04	0.3	0.03	− 0.002 to 0.06	0.065	0.068
Perceived barriers	− 0.21	− 0.24 to − 0.19	− 0.51	< 0.001	− 0.14	− 0.18 to − 0.11	− 0.36	< 0.001
Perceived benefits	0.2	0.17 to 0.23	0.4	< 0.001	0.07	0.03 to 0.11	0.14	0.001
Fatalism	− 0.4	− 0.44 to − 0.34	− 0.46	< 0.001	− 0.16	− 0.23 to − 0.09	− 0.19	< 0.001
Cues to action	0.025	0.009 to 0.04	0.11	0.002	0.01	− 0.01 to 0.04	0.04	0.4
Recognition of clinical symptom of the disease	− 0.016	− 0.1 to 0.06	− 0.01	0.7	0.07	0.00 to 0.13	0.06	0.051
Gender = men–women	− 0.13	− 0.086 to − 0.18	− 0.2	< 0.001	− 0.06	− 0.08 to − 0.04	− 0.42	< 0.001
Place of residence = urban–rural	− 0.09	− 0.15 to 0.034	− 0.11	0.002	− 0.06	0.03 to 0.09	0.24	< 0.001
Age	− 0.01	− 0.01 to 0.02	− 0.02	0.55	0.001	− 0.005 to 0.01	− 0.014	0.76

## Discussion

The results of the study indicated that rate of adherence to preventive behaviors from COVID-19 was at a desirable level. Preventive behaviors such as observing the etiquette of coughing and sneezing, washing hands for at least 20 s, not kissing others, observing at least one meter distance from others, not leaving home except when necessary, not touching nose and face by hands, not taking a mobile phone with us out of house, and washing hands with soap and water as soon as arriving home were at proper levels. Results of a study in Hong Kong of China also indicated that more than 77% of participants reported good health performance for COVID-19 [16].

Gender was an important variable affecting the preventive behaviors, so that women showed better observance than men probably since they had greater motivation for health than men. In studies on breast cancer screening behaviors, the health motivation was confirmed as an independent variable [17–19]. In a study by Lau et al. on the pandemic of H1N1 in women and men in Hong Kong of China, women had better performance than men in the prevention of the disease [20]. Moreover, people living in cities showed better performance against the disease than villagers probably due to the difference in their literacy levels.

Perceived barriers and fatalistic beliefs were also inversely related to the preventive behaviors from COVID-19. Therefore, the rate of adherence to preventive behaviors increased by reducing perceived barriers and fatalistic beliefs. However, the impact of perceived barriers was greater than fatalistic beliefs. The perceived barriers are important and effective constructs of the health belief model because the individuals should overcome barriers to behaviour despite their inner desire to engage in preventive behavior. Excessive barriers can be deterrents and prevent the creation of desired health behaviors. In the present study, the participants had fewer perceived barriers to preventive individual behaviors, such as hand washing, but they were strongly influenced by environmental barriers such as shortage of masks, alcohol pads, and disinfectants. Shortage of mask has been observed in most regions of world due to the pandemic of COVID-19 [21–24] and the issue was also observed in the present study. Majority of Chinese prefer to wear masks to protect themselves from COVID-19 [15]. The shortage of masks leads to panic buying [25] and caused anxiety and depression among the Chinese [26].

Providing masks and other disinfectants and overcoming the environmental barriers can be effective in increasing the individuals' adherence to these preventive behaviors. The existence of high perceived self-efficacy is an important factor in overcoming the perceived barriers; and it was an effective variable in adopting preventive behaviors from COVID-19 in the present study. Self-efficacy is defined as the level of trust and confidence in overcoming barriers to a healthy behavior. According to the health belief model, individuals should have an appropriate level of self-efficacy to overcome barriers to behavior [27].

Fatalistic beliefs constitute a theory based on which people believe that events are controlled by external forces and humans have no power over them and can no longer influence them; and they are considered greatly as barriers to screening and preventive behaviors for cancers. They are more common in poor people, racial and ethnic minorities, and low-literate people [28–33]. In the present study, the participants' fatalistic beliefs were low due to high levels of education and high urbanization. On the other hand, fatalistic behaviors have been studied and confirmed in diseases such as cancer, but COVID-19 is an infectious disease; and the process of its infection, like cancers, is multifactorial and sometimes unknown; and its cause is known to be a single virus. Perhaps this has also contributed to the lack of fatalistic beliefs in the participants.

Perceived benefits were other factors in predicting preventive behaviors from the disease. In other words, the individuals perform better by increasing the perceived benefits. Having perceptions such as effects of regular hand washing, use of personal protective equipment such as masks, and disposable gloves can lead to high perceived benefits, and they are thus strong motivations for taking preventive measures against this disease.

In the study, the perceived susceptibility and severity did not show any significant relationship in predicting the preventive behaviors from COVID-19 despite the fact that the significance level of perceived severity was 0.688 and close to the significance level. In general, the perceived threat construct was an important variable in taking preventive measures, so that the individuals should consider themselves susceptible to this disease and consider the severity of this disease to be dangerous. Results of a research by Li et al. also indicated that high perceived severity increased negative emotions, higher cellphone use, and caution in COVID-19 [34]. Furthermore, Kwok et al. investigated the early stages of COVID-19 in Hong Kong of China and found that the individuals had higher perceived susceptibility and severity of COVID-19, so that 89% said that they were at risk for COVID-19 and 97% said that COVID-19 had severe symptoms [16]. In the above studies, other constructs of the health belief model were not included in the study. Considering two options, I completely agree and agree, in the present study, 70.3% of participants considered themselves susceptible to coronavirus; and 72.6% considered the disease dangerous in the case of its perceived severity. In general, the perceived threat to COVID-19 is greater than H7N9 and SARS [35, 36]. Some studies indicate that the individuals, who knew themselves less susceptible to the disease, consider it a severe and dangerous disease [37, 38].

The research had three limitations: first, the data were collected from the digital space due to specific conditions caused by limitations of the disease; hence, it did not allow for random sampling to select individuals. Second, some people such as the elderly or low-income people might not have access to smart phones and not be evaluated. Third, the individuals' performance was based on self-reporting that should be considered in the data generalization. Fourth, this study did not explore the occupation of the participants. The participants might include healthcare professionals that have different preventive health behaviors [39, 40].

## Conclusions

The research results indicated that female gender, perceived barriers, perceived self-efficacy, fatalistic beliefs, perceived benefits, and living in the city respectively had the highest predictive power of preventive behaviors from COVID-19. Therefore, it is necessary to perform interventions to increase awareness in men to promote health behaviors. Inducing the benefits of preventative behaviors increases the perceived self-efficacy, and thus overcomes the barriers to preventive behaviors from COVID-19. It is suggested decreasing the fatalistic beliefs and paying more attention to people living in rural areas in order to promote preventive behaviors.

## Data Availability

The datasets used and/or analysed during the current study are available from the corresponding author on reasonable request.

## Abbreviations

COVID-19: Coronavirus disease 2019
SARS-CoV-2: Severe acute respiratory syndrome coronavirus 2
H1N1: Hemagglutinin type 1 and Neuraminidase type 1
HBM: Health belief model
RMSEA: Root mean square error of approximation
CFI: Comparative fit index
TLI: Tucker–Lewis index
SRMSR: Standardized root mean square residual
NOVA: Analysis of variance
SPSS: Statistical package for the social sciences
